# The role of drug vendors in improving basic health-care services in Nigeria

**DOI:** 10.2471/BLT.15.154666

**Published:** 2016-02-03

**Authors:** Jenny Liu, Lisa M Prach, Emily Treleaven, Mara Hansen, Jennifer Anyanti, Temple Jagha, Vince Seaman, Olufemi Ajumobi, Chinwoke Isiguzo

**Affiliations:** a Institute for Health and Aging, Social and Behavioral Sciences, University of California - San Francisco, 3333 California Street (Suite 340), San Francisco, California, CA 94118, United States of America (USA).; b Bill & Melinda Gates Foundation, Seattle, Washington, USA.; c Society for Family Health, Abuja, Nigeria.; d Partners for Human Research Empowerment and Development, Abuja, Nigeria.; e National Malaria Elimination Programme, Abuja, Nigeria.

## Abstract

**Objective:**

To characterize patent and proprietary medicine vendors and shops in Nigeria and to assess their ability to help improve access to high-quality, primary health-care services.

**Methods:**

In 2013 and 2014, a census of patent and proprietary medicine shops in 16 states of Nigeria was carried out to determine: (i) the size and coverage of the sector; (ii) the basic characteristics of shops and their staff; and (iii) the range of products stocked for priority health services, particularly for malaria, diarrhoea and family planning. The influence of the medical training of people in charge of the shops on the health-care products stocked and registration with official bodies was assessed by regression analysis.

**Findings:**

The number of shops per 100 000 population was higher in southern than in northern states, but the average percentage of people in charge with medical training across local government areas was higher in northern states: 52.6% versus 29.7% in southern states. Shops headed by a person with medical training were significantly more likely to stock artemisinin-based combination therapy, oral rehydration salts, zinc, injectable contraceptives and intrauterine contraceptive devices. However, these shops were less likely to be registered with the National Association of Patent and Proprietary Medicine Dealers and more likely to be registered with the regulatory body, the Pharmacist Council of Nigeria.

**Conclusion:**

Many patent and proprietary medicine vendors in Nigeria were medically trained. With additional training and oversight, they could help improve access to basic health-care services. Specifically, vendors with medical training could participate in task-shifting interventions.

## Résumé

**Objectif:**

Caractériser les vendeurs et magasins de spécialités pharmaceutiques et de médicaments brevetés au Nigéria et évaluer leur capacité de contribuer à l'amélioration de l'accès à des services de soins primaires de qualité.

En 2013 et 2014, un recensement des magasins de spécialités pharmaceutiques et de médicaments brevetés a été réalisé dans 16 États du Nigéria afin de déterminer: (i) la taille et l'offre de ce secteur; (ii) les caractéristiques de base des magasins et de leur personnel; et (iii) la gamme de produits en stock pour les soins prioritaires, en particulier pour le paludisme, la diarrhée et la planification familiale. L'influence de la formation médicale des personnes responsables des magasins sur les produits en stock et l'inscription auprès d'organismes officiels a été évaluée au moyen d'une analyse de régression.

Le nombre de magasins pour 100 000 habitants était plus élevé dans les États du Sud que dans ceux du Nord, mais le pourcentage moyen de personnes chargées de la formation médicale dans les zones de gouvernement local était supérieur dans les États du Nord: 52,6% contre 29,7% dans les États du Sud. Les magasins dirigés par une personne ayant une formation médicale étaient sensiblement plus nombreux à avoir en stock des associations médicamenteuses comportant de l'artémisinine, des sels de réhydratation orale, du zinc, des contraceptifs injectables et des dispositifs intra-utérins. Cependant, ces magasins étaient moins nombreux à être inscrits auprès de l'Association nationale des distributeurs de médicaments brevetés et de spécialités pharmaceutiques et plus nombreux à l'être auprès de l'organisme de règlementation, le Conseil des pharmaciens du Nigéria.

**Conclusion:**

De nombreux vendeurs de spécialités pharmaceutiques et de médicaments brevetés au Nigéria avaient reçu une formation médicale. Un contrôle et une formation complémentaire leur permettraient de contribuer à améliorer l'accès aux services médicaux de base. Les vendeurs ayant une formation médicale pourraient notamment participer aux interventions de transfert des tâches.

## Resumen

**Objetivo:**

Caracterizar los proveedores y establecimientos de medicamentos patentados y de venta libre en Nigeria y evaluar su capacidad de ayudar a mejorar el acceso a servicios sanitarios primarios de alta calidad.

**Métodos:**

En 2013 y 2014, se llevó a cabo un censo de los establecimientos de medicamentos patentados y de venta libre de 16 estados de Nigeria con el objetivo de de determinar: (i) el tamaño y cobertura del sector; (ii) las características básicas de los establecimientos y el personal; y (iii) la gama de productos destinados a servicios sanitarios prioritarios, en concreto para la malaria, la diarrea y la planificación familiar. Un análisis de regresión evaluó la influencia de la formación médica de las personas a cargo de los establecimientos de productos sanitarios almacenados y el registro en los organismos oficiales.

**Resultados:**

El número de establecimientos por cada 100 000 habitantes era mayor en los estados del sur que en los del norte, aunque el porcentaje medio de personas con formación médica en ámbitos gubernamentales locales era superior en los estados del norte: un 52,6% frente a un 29,7% en los estados del sur. Los establecimientos dirigidos por una persona con formación médica tenían almacenados más productos para tratamientos combinados basados en la artemisinina, sales de rehidratación oral, zinc, anticonceptivos inyectables y dispositivos anticonceptivos intrauterinos. No obstante, era menos probable que estos establecimientos estuvieran registrados en la Asociación Nacional de Comerciantes de Medicamentos Patentados y de Venta Libre, pero sí en el organismo regulador, el Consejo de Farmacéuticos de Nigeria.

**Conclusión:**

Se proporcionó formación médica a varios proveedores de medicamentos patentados y de venta libre en Nigeria. Con mayor formación y supervisión, podrían ayudar a mejorar el acceso a servicios sanitarios básicos. En concreto, los proveedores con formación médica podrían participar en intervenciones de rotación de tareas.

## ملخص

**الغرض:**

توصيف منافذ ومتاجر بيع الدواء التي تحمل اسم الشركات الدوائية أو المسجلة لحسابها في نيجيريا وتقييم قدرتها على المساعدة في تحسين الوصول إلى خدمات الرعاية الصحية الأساسية وعالية الجودة.

**الطريقة:**

تم في عامي 2013 و2014 إجراء تعداد لمتاجر بيع الأدوية التي تحمل اسم الشركات الدوائية أو المسجلة لحسابها في 16 ولاية في نيجيريا لتحديد ما يلي: (أ) حجم القطاع والنطاق الذي يغطيه؛ و(ب) الخصائص الأساسية لمتاجر بيع الأدوية والموظفين العاملين بها؛ و(جـ) مجموعة المنتجات المخزنة لاستخدامها في الخدمات الصحية ذات الأولوية، وخاصة لمكافحة الملاريا والإسهال وتنظيم الأسرة. تم تقييم تأثير التدريب الطبي للموظفين المسؤولين عن إدارة متاجر بيع الدواء على مخزون منتجات الرعاية الصحية والتسجيل لدى الجهات الرسمية عن طريق تحليل التحوف.

**النتائج:**

كان عدد متاجر بيع الدواء في نطاق يبلغ 100,000 من السكان في الولايات الجنوبية أكبر مما كان عليه في الولايات الشمالية، ولكن كان متوسط النسبة المئوية للأشخاص الذين يديرون المتاجر ولديهم خبرة التدريب الطبي في المناطق الحكومة المحلية أعلى في الولايات الشمالية: 52.6% في مقابل 29.7% في الولايات الجنوبية. كانت المتاجر التي يديرها شخص حاصل على التدريب الطبي الأكثر احتمالاً في تخزين العلاج المشترك المرتكز على الأرتيميزينين، وأملاح الإماهة الفموية، والزنك، ومانعات الحمل القابلة للحقن، ووسائل منع الحمل داخل الرحم. ومع ذلك، كانت هذه المتاجر أقل احتمالاً أن تكون مسجلة لدى الجمعية الوطنية لمنافذ توزيع الأدوية التي تحمل اسم الشركات الدوائية أو المسجلة لحسابها والأرجح أن تكون مسجلة لدى الهيئة التنظيمية، مجلس الصيادلة في نيجيريا.

**الاستنتاج:**

كان العديد من المسؤولين عن إدارة منافذ بيع الأدوية التي تحمل اسم الشركات الدوائية أو المسجلة لحسابها حاصلين على التدريب الطبي. ومن خلال المزيد من التدريبات الإضافية والإشراف، يمكنهم المساعدة في تحسين الوصول إلى خدمات الرعاية الصحية الأساسية. وعلى وجه التحديد، يمكن للقائمين على منافذ بيع الدواء الحاصلين على التدريب الطبي المشاركة في تدخلات تحويل المهام.

## 摘要

**目的:**

旨在分析尼日利亚专利和专有药品供应商和商店的特征以及评估他们在提供高质量初级医疗卫生服务方面的能力。

**方法:**

2013 年至 2014 年中，尼日利亚对其 16 个州展开了专利和专有药品商店普查，以确定：(1) 该行业部门的规模和覆盖面；(2) 商店及其员工的基本特征；和 (3) 为主要卫生服务，尤其是疟疾、腹泻以及生育计划存储的产品范围。采用回归分析法分析了针对商店负责人员进行医疗培训对医疗卫生产品存储和官方机构注册的影响。

**结果:**

南部地区每十万人的商店数高于北部地区的数量，但是在北部地区，整个当地政府区域，负责医疗培训的人员平均比例较高：为 52.6%，而在南部地区为 29.7%。由经过医疗培训的人员负责的商店存储青蒿素为基础的联合疗法、经口补液盐、锌、注射式避孕药和子宫环的可能性显著更大。然而，这些商店在国家专利和专有医药经销商处注册的可能性更小，而在尼日利亚药剂师理事会这一监管机构注册的可能性更大。

**结论:**

尼日利亚许多专利和专有药品供应商都经过医疗培训。通过额外的培训和监督，他们能够帮助改善基本医疗卫生服务的普及率。具体而言，经过医疗培训的供应商能够参与重新分工的干预措施。

## Резюме

**Цель:**

Охарактеризовать продавцов патентованных лекарственных средств и соответствующие магазины в Нигерии и дать оценку их способности увеличить доступ к высококачественному первичному медико-санитарному обслуживанию.

**Методы:**

В 2013 и 2014 гг. была проведена перепись магазинов, реализующих патентованные лекарственные средства, в 16 штатах Нигерии с целью определить: (i) размер и охват сектора, (ii) основные характеристики магазинов и их персонала и (iii) ассортимент имеющихся в наличии товаров для оказания первичной медико-санитарной помощи при лечении малярии, диареи и планировании семьи. Для оценки того, как влияет наличие медицинского образования у заведующих магазинами на наличие товаров медицинского назначения и постановку на учет в официальных органах, использовался регрессионный анализ.

**Результаты:**

Количество магазинов на 100 000 жителей было больше в южных штатах, чем в северных, однако в среднем процент заведующих магазином с медицинским образованием на различных территориях местного управления был гораздо выше в северных штатах: 52,6% по сравнению с 29,7% в южных штатах. В магазинах, которыми заведовали лица с медицинским образованием, вероятность наличия в продаже комбинированной терапии на базе артемизинина, солей для пероральной регидратации, цинка, инъецируемых контрацептивов и внутриматочных противозачаточных средств была значительно выше. Однако эти магазины проходили регистрацию с меньшей вероятностью в Национальной ассоциации торговцев патентованными лекарственными средствами и с большей вероятностью в регулятивном органе — Совете фармацевтов Нигерии.

**Вывод:**

Большинство продавцов патентованных лекарственных средств в Нигерии получили медицинское образование. При дополнительной подготовке и надзоре они могли бы поспособствовать увеличению доступа к базовому медико-санитарному обслуживанию. В частности, продавцы с медицинским образованием могли бы принимать участие в мероприятиях по перераспределению обязанностей.

## Introduction

Both specialized emergency care and basic health-care services depend on the presence of a well-staffed and well-trained, health-care workforce. Yet, despite the high burden of disease in sub-Saharan Africa, many countries have a shortage of health workers that is projected to persist well into the future.^1^ New strategies for developing a robust health-care workforce are needed to help achieve universal health coverage and health equity.

Studies have shown that front-line health workers, including community health workers, can improve access to – and the equity of – health services.^2^^,^^3^ Community access to trained health workers and essential health-care products are core elements of patient-centred health-care systems in places without access to formal health care.^4^ In sub-Saharan Africa, people often seek care from drug vendors (i.e. patent and proprietary medicine vendors) for common but potentially deadly illnesses, such as malaria and diarrhoea.^5^ Although vendors are not always recognized as front-line health workers, they provide the first and the main point of care in many communities. In some settings, training drug vendors to provide high-quality basic services, such as the treatment of common childhood illnesses and malaria, may offer a cost-effective way of delivering community-based health programmes.^6^

Nigeria, which faces many health-care challenges, is a prime example of a country where drug vendors could supplement the health-care workforce and improve access to basic primary care currently beyond the reach of many people. Drug vendors, who are not required to have formal pharmacy training but who sell prepackaged, over-the-counter pharmaceutical products on a retail, for-profit basis,^7^ are the main access points for many health-care products and services.^8^ They are also consulted for advice and diagnosis, particularly by poor, rural and marginalized people with limited access to formal health services.^9^^–^^12^ Previous study has shown that some of the vendors investigated (53/250) had some medical training.^13^

There is no reliable estimate of the number of drug vendors in Nigeria or of their locations and the services they offer – basic information needed to understand how this sector could be better engaged. This is partly because vendors often fail to register with the Pharmacist Council of Nigeria, the official regulatory body for both pharmacies and patent and proprietary medicine vendors.^14^ Rather, vendors prefer to register with their professional association, the National Association of Patent and Proprietary Medicine Dealers, which provides support such as monitoring the types of products sold, facilitating education and training and giving business and financial assistance.^15^^,^^16^ However, this organization does not have a regulatory mandate.

Any national plan or policy to deliver quality-assured, health-care services and products through patent and proprietary medicine vendors’ shops depends on knowledge of the characteristics, stocking practices and coverage of these shops. We conducted a census of shops in 16 states in Nigeria to: (i) document the size and coverage of the patent and proprietary medicine sector; (ii) describe the basic characteristics of the shops and their workers; and (iii) assess the range of products stocked, including those for common illnesses, such as malaria and diarrhoea, as well as for priority health-care needs, such as family planning. We also examined the relationship between the products stocked and the shop’s registration with official bodies and the medical training of the person in charge.

## Methods

In 2013 and 2014, we conducted a census of all patent and proprietary medicine vendors’ shops that could be identified in 16 of the 36 Nigerian states: Akwa-Ibom, Bauchi, Delta, Edo, Jigawa, Kano, Katsina, Kebbi, Kogi, Kwara, Lagos, Ogun, Oyo, Rivers, Sokoto and Zamfara. The census collected information on the basic characteristics of the shops, their owners and the health-care products stocked. Since cultural and sociodemographic factors influence care-seeking behaviour and shape the way communities interact with vendors,^10^^,^^17^^,^^18^ we selected states across both northern and southern regions of the country. We excluded states in the north-east because of security concerns.

The main fieldwork was conducted in May 2013, with data verification and quality assurance continuing until March 2014. We hired 140 field staff and six supervisors. Field staff underwent three days of training on standard operating procedures, the survey protocol, data collection tools and the use of Global Positioning System (GPS) receivers for capturing geospatial data. Two field staff were assigned to each local government area – the administrative level below the state level. First, approval for the study was obtained from the local branch of the National Association of Patent and Proprietary Medicine Dealers, which also provided a list of members’ outlets in the area. Then, field staff visited all patent and proprietary medicine shops that could be located. They also included any other shops observed incidentally or identified by shop workers or community residents.

The geographical coordinates of each shop were recorded and a brief questionnaire was administered to the person in charge. If the person in charge was unavailable, field staff returned until he or she could be interviewed. The questionnaire asked about the shop’s registration, the personal details of the person in charge (e.g. professional and educational qualifications) and current stocks of health-care products, particularly those for family planning, malaria, pneumonia and diarrhoea. The person in charge was regarded as having had medical training if he or she reported having a formal qualification as a medical doctor, nurse, midwife or pharmacist or having completed a training programme as a community health extension worker or a two- or three-year clinical training programme as a junior community health extension worker.^19^ In each state, two supervisors supported and managed field staff and monitored data quality. Data were recorded and checked for quality using CSPro (United States Census Bureau, Suitland, United States of America), SPSS (SPSS Inc., Chicago, USA) and ArcGIS v. 10.1 (Esri, Redlands, USA).

Statistical and spatial analyses were conducted using Stata v. 13.1 (StatCorp. LP, College Station, USA) and QGIS v. 2.8 (QGIS Development Team), respectively. The main outcomes were: (i) current stocks of recommended drugs to treat malaria (i.e. any brand of artemisinin-based combination therapy) and diarrhoea (i.e. oral rehydration salts and zinc); (ii) current stocks of family planning products, including any brand of oral contraceptive pill, injectable contraceptives, emergency contraception and intrauterine contraceptive devices; and (iii) registration with the National Association of Patent and Proprietary Medicine Dealers or the Pharmacist Council of Nigeria. A shop was regarded as stocking a particular product if the respondent indicated that the product was available when read a checklist of types and brands of products. Any products in stock but not on the checklist were noted and reclassified on a case-by-case basis.

We also recorded the geographical location of the shop (i.e. the state and whether in an urban or rural location), the number of staff, the estimated number of customers per day and the medical training of the person in charge. We calculated the number of shops per 100 000 population in each local government area – the smallest geographical unit for which population data were available – using 2006 national census estimates adjusted for population growth to 2012.^20^ In addition, for each local government area, we calculated the percentage of people in charge of the shops who had medical training. Both parameters were shown graphically on maps.

Bivariate and multivariate analyses were used to examine the relationship between current stocks and registration and the medical training of the person in charge and logistic regression analysis was used to calculate odds ratios for the influence of medical training on these outcomes. To account for possible sources of confounding, we controlled for the location of the shop (i.e. the state and urban or rural location), the number of staff employed and the reported number of customers per day. To estimate standard errors and enable statistical inference, we used a bootstrap method with 100 repetitions to draw repeated samples from the census data for all regression models. The study was approved by the National Health Research Ethics Committee of Nigeria, Federal Ministry of Health.

## Results

**Fig. 1:**
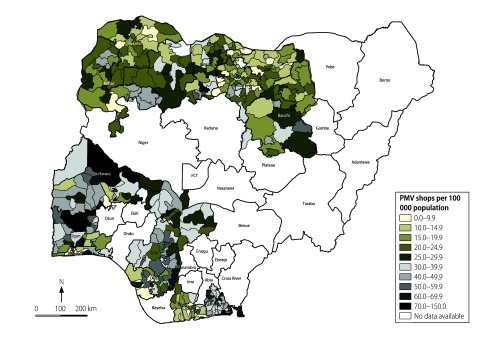
Patent and proprietary medicine shops per 100 000 population, 16 states in Nigeria, 2013–2014

Note: The number of shops per 100 000 population is shown for each local government area included in the census.

Source: Shape files were obtained from DIVA-GIS.

Table 1 lists the characteristics of the 20 642 shops identified in the 16 states. The number in each state ranged from 728 in Sokoto to 3044 in Oyo. Fig. 1 shows the number of patent and proprietary medicine shops per 100 000 population in each local government area. On average, there were 24 shops per 100 000 population in all areas combined: the average was 19 per 100 000 in the northern states surveyed and 31 per 100 000 in the southern states. In addition, 42.7% (8399) of shops were located in rural areas. 

**Table 1:** Census of patent and proprietary medicine shops, 16 states in Nigeria, 2013–2014

Characteristic	No. (%) of shops, (*n* = 20 642)^a^
Product stocked	
Any artemisinin-based combination therapy	16 966 (82.2)
Oral rehydration salts^b^	17 662 (92.1)
Zinc^b^	3 671 (19.1)
Any oral contraceptive	13 941 (67.5)
Injectable contraceptive	4 469 (21.7)
Emergency contraception	5 208 (25.2)
Intrauterine contraceptive device^c^	566 (2.9)
**Registration^d^**	
National Association of Patent and Proprietary Medicine Dealers	15 619 (81.4)
Pharmacist Council of Nigeria	2 565 (13.4)
**Medical training of person in charge**	
No medical training	13 491 (65.4)
Some medical training	7 151 (34.6)
Community health extension worker	2 656 (12.9)
Nurse or midwife	2 554 (12.4)
Junior community health extension worker	1 090 (5.3)
Pharmacist	492 (2.4)
Doctor	226 (1.1)
Other health-care worker	133 (< 1.0)
**Other staff^e^**	
0	5 104 (25.5)
1	7 013 (35.0)
2	5 775 (28.8)
≥ 3	2 147 (10.7)
**Customers per day (estimated)^f^**	
0–10	4 190 (22.0)
11–20	5 589 (29.3)
21–30	4 756 (25.0)
≥ 31	4 523 (23.7)
**Location^g^**	
Urban	11 258 (57.3)
Rural	8 399 (42.7)
**State**	
Northern states combined	7 532 (36.5)
Bauchi	1 061 (5.1)
Jigawa	770 (3.7)
Kano	1 695 (8.2)
Katsina	1 522 (7.4)
Kebbi	934 (4.5)
Sokoto	728 (3.6)
Zamfara	822 (4.0)
Southern states combined	13 110 (63.5)
Akwa-Ibom	1 714 (8.3)
Delta	1 394 (6.7)
Edo	1 305 (6.3)
Kogi	1 088 (5.3)
Kwara	1 002 (4.8)
Lagos	1 374 (6.7)
Ogun	1 271 (6.2)
Oyo	3 044 (14.7)
Rivers	918 (4.5)

^a^ Total number of shops with data available except where otherwise indicated.

^b^ Data were available for only 19 179 shops.

^c^ Data were available for only 19 160 shops.

^d^ Data were available for only 19 192 shops.

^e^ Data were available for only 20 039 shops.

^f^ Data were available for only 19 058 shops.

^g^ Data were available for only 19 657 shops.

**Fig. 2:**
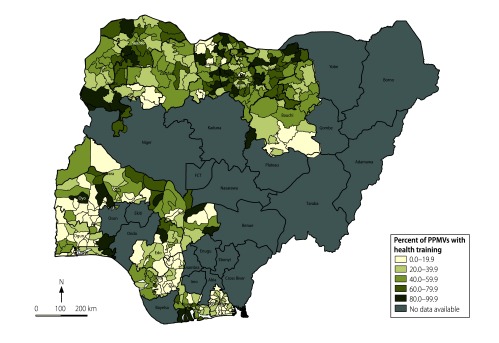
Proportion of people in charge of patent and proprietary medicine shops that have medical training, 16 states in Nigeria, 2013–2014

Notes: Percentages are shown for each local government area included in the census. Medical training included self-reported formal training to qualify as a doctor, midwife, nurse, pharmacist, community health extension worker or junior community health extension worker.

Source: Shape files were obtained from DIVA-GIS.

In 7151 shops (34.6%), the person in charge reported having had some form of medical training. Fig. 2 shows the percentage of people in charge with medical training by local government area. The average percentage was higher across local government areas in northern than in southern states: 52.6% versus 29.7%, respectively (Fig. 2). In the south, Akwa-Ibom, Oyo and Delta states had the lowest average percentages with medical training across local government areas (17.4%, 23.8% and 24.9%, respectively), whereas, in Kogi and Rivers states, the figure was over 40% across local government areas.

The health-care products the drug vendor had in stock varied by type. For common illnesses, 82.2% (16 966) of shops stocked an artemisinin-based combination therapy and 92.1% (17 662) stocked oral rehydration salts but only 19.1% (3 671) carried zinc. For family planning, 67.5% (13 941) stocked oral contraceptive pills, 21.7% (4 469) had injectable contraceptives and 25.2% (5 208) had emergency contraception but only 2.9% (566) stocked intrauterine contraceptive devices. Overall, 81.4% (15 619) of shops were registered with the National Association of Patent and Proprietary Medicine Dealers, whereas only 13.4% (2 565) were registered with the Pharmacist Council of Nigeria (Table 1).

The bivariate analysis found a significant association between medical training and stocks of seven products and registration status. In the multivariate analysis, the estimated effect size was smaller for most outcomes but all associations except two (i.e. oral contraceptive and emergency contraception stocks) remained significant. Consequently, after controlling for confounders, there were significant associations between the medical training of the person in charge and the likelihood that a shop would stock artemisinin-based combination therapy, oral rehydration salts, zinc, injectable contraceptives and intrauterine contraceptive devices. In addition, shops headed by a person with medical training were significantly more likely to be registered with the Pharmacist Council of Nigeria and significantly less likely to be registered with the National Association of Patent and Proprietary Medicine Dealers (Table 2).

**Table 2:** Association of any medical training of person in charge of a patent and proprietary medicine shop with current stock and registration, 16 states in Nigeria, 2013–2014

Outcome	No. (%) of people in charge of shop with any medical training			Likelihood of outcome associated with medical training, OR (95% CI)^a^	
	Yes	No		Bivariate analysis	Multivariate analysis^b^
**Product stocked**					
Any artemisinin-based combination therapy	6 513 (38.4)	10 453 (61.6)		2.97 (2.71–3.25)	1.57 (1.39–1.76)
Oral rehydration salts	6 681 (37.8)	10 981 (62.2)		1.47 (1.32–1.62)	1.16 (1.01–1.32)
Zinc	1 572 (42.8)	2 099 (57.2)		1.34 (1.26–1.43)	1.12 (1.03–1.23)
Any oral contraceptive	5 048 (36.2)	8 893 (63.8)		1.24 (1.17–1.31)	0.95 (0.89–1.02)
Injectable contraceptive	2 261 (50.6)	2 208 (49.4)		2.36 (2.21–2.52)	1.37 (1.28–1.48)
Emergency contraception	1 701 (32.7)	3 507 (67.)		0.89 (0.84–0.95)	1.06 (0.99–1.14)
Intrauterine contraceptive device	304 (53.7)	262 (46.3)		2.00 (1.64–2.44)	2.07 (1.71–2.50)
**Registration**					
National Association of Patent and Proprietary Medicine Dealers	5 506 (35.3)	10 113 (64.7)		0.65 (0.61–0.70)	0.87 (0.79–0.95)
Pharmacist Council of Nigeria	1 137 (44.3)	1 428 (55.7)		1.41 (1.32–1.52)	1.50 (1.34–1.67)

CI: confidence interval; OR: odds ratio.

^a^ ORs were estimated using logistic regression. Standard errors for generating confidence intervals were estimated using bootstrap methods with 100 repetitions. Reference category is person without medical training in charge of the patent and proprietary medicine shop.

^b^ The multivariate analysis controlled for the state in which the shop was located, the estimated number of customers per day, urban or rural location and the number of staff members.

There were no consistent relationships between health qualifications and urban or rural location, the number of staff employed and the estimated number of customers per day.

## Discussion

Our census of patent and proprietary medicine vendors’ shops revealed two important features of the sector in Nigeria that could be used by policy-makers to expand access to primary care services. First, shops were plentiful in many parts of the country, particularly in southern states. Moreover, in southern states, there were more shops than public or private health-care facilities, including health posts, dispensaries, clinics and hospitals:^21^ 31 versus 24 per 100 000 population, respectively. In northern states, the density of shops was comparable to the density of health-care facilities.^21^ Thus, these shops may be more accessible than health-care facilities in many places.

Second, many drug vendors have undergone formal medical training. We found that the proportion of vendors with medical training was higher in northern states, where health outcomes and service utilization lags behind the rest of the country.^8^ These vendors may be able to deliver high-quality health services and thereby complement the existing health-care infrastructure. In fact, vendors with medical training were more likely to stock recommended drugs for common illnesses and longer-acting family planning products such as injectable contraceptives and intrauterine devices, whose use requires assistance from a skilled provider. Although drug vendors are currently prohibited from performing these invasive procedures,^7^ qualified health professionals, including community health extension workers, nurses, midwives and doctors, may do so.^22^ Drug vendors with health professional qualifications must abide by the guidelines for patent and proprietary medicine vendors when conducting business in their shops but may operate within the scope of their professional training outside their shops.

The suggestion that drug vendors could deliver basic health services to underserved populations must, however, be tempered by concerns about quality assurance. Several studies have shown that vendors have poor knowledge of health, health care and drugs, often dispense drugs improperly and may provide services beyond their legal scope of practice.^23^ Moreover, one study found that there may be no difference in knowledge or stocking practices between vendors with and without medical training,^13^ which suggests that training may not ensure high-quality care.

More effective engagement of drug vendors in community-based health services depends on recognizing their role and including them in national health strategies. In 2014, the Government of Nigeria issued a task-shifting and task-sharing policy for essential health-care services in Nigeria,^22^ which addressed shortages in the availability of health workers needed to deliver essential health services. The policy called for an increase in the capacity of people in the community, including drug vendors, to provide treatment, counselling and referral for some reproductive and maternal and child health services, including malaria treatment. Although the services that can be provided by vendors are limited at present, there is an aspiration to delegate more basic health services to lower level cadres (B Orji and M Oyinbo, personal communication, 8 September 2015). The implementation of effective training and accreditation programmes for drug vendors will have to reconcile the duties of front-line health workers specified in the task-shifting and task-sharing policy with national treatment guidelines. The implementation will also have to clarify the legal scope of practice regarding service delivery in nonclinical settings for drug vendors who may also be health professionals. In addition, establishing a holistic approach to monitoring and regulation should be considered. The approach should engage drug vendors who are able and willing to provide a greater range of basic health services at the required quality standard. Such services could include simple diagnostic testing, such as rapid diagnostic tests for malaria, giving injections and dispensing antibiotics to treat pneumonia in accordance with integrated community case management of childhood illness.^24^

Pilot studies have shown that drug vendors in sub-Saharan Africa were capable of delivering additional services, such as rapid diagnostic tests for malaria.^25^^–^^27^ In Nigeria, delivery of such tests has increased the precision and quality of malaria case management.^6^^,^^28^^–^^30^ These findings support a limited expansion of drug vendors’ activities. In addition, evidence from elsewhere in sub-Saharan Africa demonstrates how drug vendors can be successfully recruited for primary care. In Uganda, an intervention that involved training private drug vendors to provide integrated community case management resulted in the appropriate treatment of malaria, pneumonia and diarrhoea in most cases.^6^^,^^26^^,^^31^ The accredited drug dispensing outlets programme in the United Republic of Tanzania demonstrated that formally integrating drug vendors into the health system increased access to affordable, high-quality medicines and services in rural areas.^32^ In Rwanda, the provision of basic diagnostic and treatment services by a large cadre of community health workers helped reduce public sector costs and improved infectious disease control.^33^ In these countries, it was found that, to be successful, efforts to engage drug vendors must take into account incentives, the establishment of a regulatory structure for training, accreditation and monitoring and the level of demand for services in the community.^6^^,^^31^^–^^33^

If drug vendors are to be effectively engaged in helping achieve public health goals, it is essential to consider establishing complementary and supportive structures. These structures will ensure a minimum level of quality, provide appropriate medical training, oversight and accountability, and strengthen links to higher-level health services. For example, the self-regulatory and peer-mentoring activities currently carried out by the National Association of Patent and Proprietary Medicine Dealers are broadly accepted by drug vendors.^16^ These activities could be recognized more formally and strengthened to improve the enforcement of quality standards. In our census, most shops were registered with the National Association of Patent and Proprietary Medicine Dealers, rather than the Pharmacist Council of Nigeria. Vendors with medical training were twice as likely to be registered with the Pharmacist Council than those without medical training, which suggests that vendors with qualifications may be more likely to follow regulatory and quality assurance guidelines.

Formalizing links between qualified drug vendors and other health professionals, including pharmacists and facility-based professionals, could improve access to prescription-only medicines and higher-level care in a complementary fashion. Such links could also increase adherence to best practice, as described in treatment guidelines, and facilitate referrals. Direct competition between accredited drug vendors and community pharmacists would probably be limited because pharmacies are concentrated in urban areas and offer a full range of over-the-counter and prescription medications. Nonetheless, cooperation between the National Association of Patent and Proprietary Medicine Dealers and the Pharmacist Council of Nigeria is required to develop mechanisms that both raise drug vendors’ standards and facilitate regulatory compliance (e.g. by increasing drug vendors’ registration with the Pharmacist Council of Nigeria). If drug vendors are to perform more advanced tasks, professional organizations of community health extension workers, doctors, nurses and midwives will need to be consulted to define parameters under which drug vendors of different trainings levels and/or professional competencies can operate.

Our study has several limitations. The states in which the census was conducted were purposively selected and are thus not representative of Nigeria as a whole. The characteristics of drug vendors in other states may be different and may exhibit variations not captured here. Yet, we sought to locate every patent and proprietary medicine shop in the 16 states. The wide geographical spread of the shops meant that we could conduct only a brief survey at each shop and, moreover, all survey responses were self-reported. Reporting bias is, therefore, a possibility. Only the GPS location of each shop could be independently verified.

The integration of patent and proprietary medicine vendors into the formal health-care system could increase access to high-quality, primary health-care services throughout Nigeria. The sector has a large capacity and recent health policy changes are supportive. Policy-makers should consider encouraging drug vendors with medical training to participate in health interventions because they may be able to offer high-quality services, particularly with appropriate training and monitoring. A formal system for registering drug vendors with a regulatory body would help support continuing medical education.
